# Effect of Different Walnut and Hazelnut Leaf Compost Treatments on Yield and Phenolic Composition of *Lactuca sativa* L.

**DOI:** 10.3390/foods12142738

**Published:** 2023-07-19

**Authors:** Aljaz Medic, Anita Solar, Metka Hudina, Robert Veberic, Tilen Zamljen

**Affiliations:** Department of Agronomy, Biotechnical Faculty, University of Ljubljana, SI 1000 Ljubljana, Slovenia; anita.solar@bf.uni-lj.si (A.S.); metka.hudina@bf.uni-lj.si (M.H.); tilen.zamljen@bf.uni-lj.si (T.Z.)

**Keywords:** allelopathy, lettuce, naphthoquinones, toxic residue, HPLC, mass spectrometry, LC-MS

## Abstract

The use of compost made from the leaves of *Juglans regia* has long been controversial because of its inhibitory effect due to the presence of juglone. Therefore, the aim of our study was to replicate the typical habits of farmers and gardeners, where the dried leaves are collected at the end of the season and placed in a composter. Then, the effects of the different treatments on the yield of the plant (lettuce), secondary metabolism, and possible toxicity of the compost of the grown plant were evaluated. The lowest yield of lettuce was obtained in soil with composted walnut and hazelnut leaves, while the highest yield was recorded in in soil with compost control, soil with composted walnut leaves and grass with the addition of composting agent and soil with composted walnut leaves with addition of composting agent. Some allelochemicals were still present in the compost but at such low levels that they did not affect yield. We suggest that dry walnut leaves and cut grass can be used for composting, while dry hazelnut leaves still contain some allelochemicals after two years that significantly inhibit plant growth and thus yield, so we would not recommend their use for composting.

## 1. Introduction

Composting is a technical process in which organic waste is microbiologically degraded to humic substances. Composting is suitable for the treatment of many solid wastes, including farm manure, sewage sludge, and especially crop residues. The goal of composting is to reduce the amount of undesirable substances such as plant and animal pathogens and organic compounds that are considered hazardous to the environment, as well as to reduce the carbon concentration and increase the concentration of plant nutrients. Composting essentially converts materials that are not suitable for land application into materials that can be used for land application [1]. In addition, the application of composted organic waste is considered efficient in terms of energy savings and solid waste disposal costs. Currently, composting is an important industry involving cooperatives and companies as well as farms and organic gardeners [2].

During the growing season, plants produce a variety of agro-waste or plant residues that can release allelochemicals into the soil. Depending on their effect on the growing plant, these allelochemicals are divided into positive ones, which stimulate plant growth, and negative ones, which inhibit growth. They can cause malformation and wilting, induce chlorosis, reduce plant vigor, hinder plant development and growth, and slow or prevent plant germination. All these may eventually lead to the complete collapse of a plant [3]. Moreover, allelochemicals can not only alter the yield of plants but also the metabolism of the plant, especially the secondary metabolism and phenolic profile [4,5]. Since phenolic compounds are considered to be the most important factor in the health benefits of fruits and vegetables besides vitamins [6], this effect is very important but still poorly studied. Phenolic compounds, in addition to their health-promoting effects (e.g., antimicrobial, antiallergenic, anti-inflammatory, and antioxidant activity) [7,8,9,10], also play an important role in the quality of fruits and vegetables by influencing their flavor, appearance, and stability [11].

The use of compost made from the leaves of *Juglans regia* L. has long been controversial, as it has been associated with its germination- and growth-inhibiting effects, due to juglone presence which was previously reported to affect germination, growth, yield, and secondary metabolism of the grown plants [4,5,12]. Previously, young compost (less than six months) from fresh walnut leaves was reported to have a negative effect on germination and growth of test plants, while older compost (nine to ten months) from fresh walnut leaves did not inhibit germination or plant growth. In addition, the nutrients of walnut leaf compost lead to higher green mass of the grown plants compared to the control [13]. However, there are very few studies on this topic, and the only study [13] used fresh leaves for compost, which is not so relevant because the leaves falling from walnut trees are usually not fresh but already dry. Since the dry leaves are usually collected at the end of the growing season and placed in a composter, the objective of our study was to replicate the typical habits of farmers and gardeners where the dried leaves are collected at the end of the season and placed in a composter. Since other plant waste is usually added to the compost but also to ensure homogeneity, the compost was mixed with various plant residues and additives to speed up composting. Then, the effects of the different treatments on the yield of the plant (lettuce), secondary metabolism, and possible toxicity of the grown plant were evaluated. These results will further improve the knowledge of compost from walnut residues and answer the question of how the compost affects the yield and phenolic profile and thus the health benefits of the plants grown in this compost.

## 2. Materials and Methods

### 2.1. Compost Preparation

**Figure 1:**
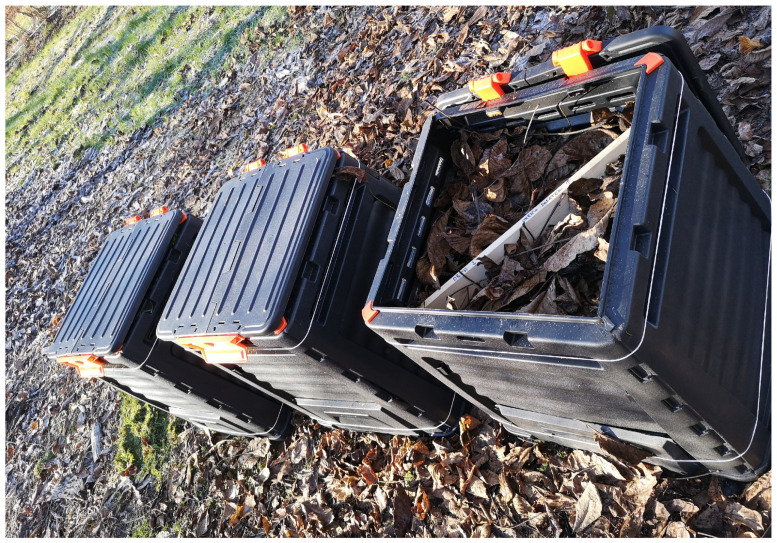
Compost bins used and divided by the wooden barriers.

Composting was performed at the Experimental Field for Nut Crops in Maribor (Slovenia; 46°34′01″ N; 15°37′51″ E; 275 m a.s.l.). All plant material was also obtained at this site. Four compost bins were used for the experiment, three of which were divided into two halves by a wooden barrier, as one half of the compost bin was used for the treatments without the composting agent and the other half was used for the treatments with the composting agent (see Figure 1). The composting agent selected was used because it is commonly used by gardeners and farmers for faster composting to see the effects on the yield of the crops grown. The composting agent used was COMPO BIO (COMPO GmbH, Münster, Germany).

For the experiment, 7 compost treatments and a control group were used. One compost bin was used to compost various plant residues commonly used by an allotment gardener or farmer (excluding grass, hazelnut, and walnut leaves), which was a positive control. The remaining three bins were divided into two parts (one with composting agent and one without) as previously described and treated as follows: walnut leaves, walnut leaves with the addition of the composting agent, walnut and hazelnut leaves (ratio 1:1), walnut and hazelnut leaves (ratio 1:1) with the addition of the composting agent, freshly cut grass and walnut and hazelnut leaves (ratio 1:1:1), and freshly cut grass and walnut and hazelnut leaves (ratio 1:1:1) with the addition of the composting agent. Composting was carried out for 2 years, as is common practice among gardeners.

After two years, the compost was carefully emptied and packed into bags and transported to the experimental field of the Department of Agronomy at the Biotechnical Faculty of the University of Ljubljana, Slovenia. There, the compost was mixed separately with soil in a 9:1 ratio for each treatment. After mixing, the mixture of soil and compost was labeled as further described in the text: K2, soil with compost control; O, soil with composted walnut leaves; O + D, soil with composted walnut leaves with addition of composting agent; O + L, soil with composted walnut and hazelnut leaves; O + L + D, soil with composted walnut and hazelnut leaves with addition of composting agent; O + T, soil with composted walnut leaves and grass; and O + T + D, soil with composted walnut leaves and grass with addition of composting agent. In addition, a control treatment with pure soil without compost was added, which is a negative control and is referred to as K1 in the text.

### 2.2. Plant Material and Growing Conditions

**Figure 2:**
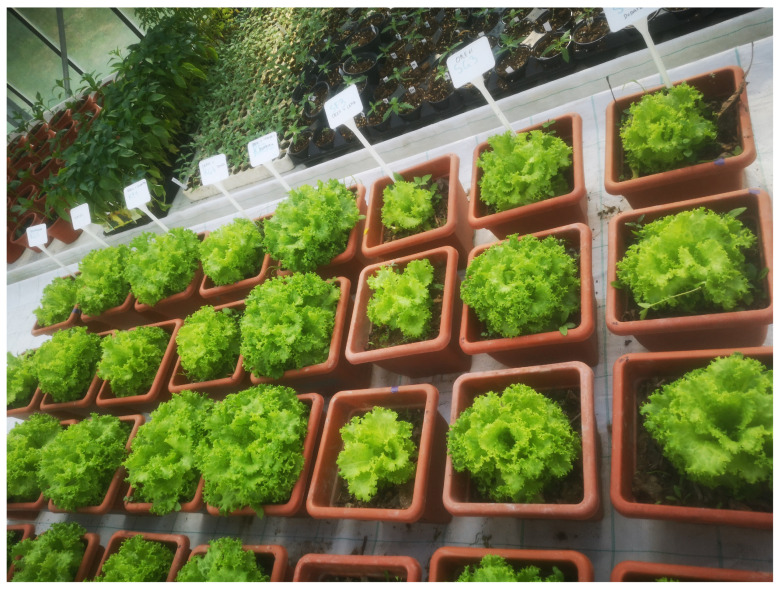
Visual representation of the experiment: Different treatments affecting the yield of the *L. sativa*.

The plants treated were lettuce (*Lactuca sativa*), as it grows quickly and is a common garden vegetable to which compost is usually added before planting. Plants were grown in a greenhouse to better control growing conditions and watered evenly every other day. Seeds were obtained from Austrosaat AG, Wien, Austria, and the cultivar chosen was ‘Tourbillon’. Plants were germinated from seeds in the greenhouse and sown in pots with standard peat substrate and repotted into soil with different compost treatments after the appearance of the third leaf (29 March 2022). After technological maturity (60 days after the seeds were planted), the plants were sampled (28 May 2022). There were 10 replicates per treatment. A total of 80 lettuce plants were used. Of these 10 plants per treatment, all 10 were weighed to determine their yield; then, 10 plants (2 plants were used per replicate) were used for metabolite analysis (Figure 2), giving 5 replicates per treatment for metabolite analysis.

### 2.3. Chemicals

The following standards were used for the identification and quantification of phenolic compounds: *p*-coumaric acid, caffeic acid, gallic acid, sinapic acid, chlorogenic acid (3-caffeoylquinic acid), cryptochlorogenic acid (4-caffeoylquinic acid), sinapic acid (Sigma-Aldrich Chemie GmbH, Buchs, Switzerland), quercetin-3-*O*-glucoside, and kaempferol-3-*O*-glucoside (Fluka Chemie GmbH, Buchs, Switzerland).

A Milli-Q water purification system from Millipore (Bedford, MA, USA) was used for the bi-distillation and purification of the water used. The acetonitrile and formic acid used as mobile phases for MS analysis were of HPLC-MS quality (Fluka Chemie GmbH). The methanol used for the extraction of the phenolic compounds was of the quality HPLC-MS (Sigma-Aldrich Chemie GmbH).

### 2.4. Sampling of the Plants and Extraction of the Phenolic Compounds

After sampling, whole plants were immersed in liquid nitrogen and lyophilized, then ground to powder using a mill (A11 basic analytical mill, IKA^®^-Werke GmbH &Co., KG, Staufen, Germany), and stored until further analysis.

The extraction was performed according to the protocol described by Medic et al. [5]. Briefly, 0.2 g of the previously lyophilized sample was extracted at a tissue-to-solution ratio of 1:30 (*w*/*v*) using an extraction medium of 80% methanol and 3% formic acid in water.

### 2.5. HPLC—Mass Spectrometry Analysis of Individual Phenolic Compounds

A tandem mass spectrometer (LTQ XL, Thermo Scientific, Waltham, MA, USA) coupled to a UHPLC (Vanquish, Thermo Scientific, Waltham, MA, USA) operated in negative ion mode and heated electrospray ionization was used for the identification of phenolic compounds. Parameters were as described by Medic et al. [5]. Data acquisition was performed using Xcalibur 2.2 software (Thermo Fischer Scientific Institute, Waltham, MA, USA).

Quantification was performed on a Vanquish UHPLC system with a diode detector set at 350 nm for flavanols and 280 nm for the other phenolic compounds. A Gemini 150 mm × 4.60 mm, 3 µm C18 column (Phenomenex, Torrance, CA, USA) was used for the separation of phenolic compounds. The solvents, column temperature, elution flow gradient, gradient, and other parameters were as described by Medic et al. [5].

Commercial standards were used wherever possible. Where these were not available, identification was based on literature data and the specific fragmentation pattern of the compounds, and quantification was performed using a similar standard as shown in Table 1.

### 2.6. Statistical Analysis

Data were compiled using R commander (package Rcmdr) version 2.7.1 (Team, R.D.C., 2008, Stanford, CA, USA) and Microsoft Excel (MS Office 2016). Five replicates (for each treatment) were performed. Data are presented as means ± standard error (SE). One-way analysis of variance (ANOVA) with Tukey tests was used to determine differences between treatments. Statistical means were calculated at a 95% confidence level to determine the significance of differences.

## 3. Results and Discussion

### 3.1. Identification of Individual Phenolic Compounds in L. sativa

A total of 18 phenolic compounds were identified in *L. sativa* based on the available literature, pseudomolecular ion [M−H]^−^, and their specific fragmentation patterns (MS^2^, MS^3^, and MS^4^). Among these 18 compounds were 10 hydroxycinnamic acids, 2 hydroxybenzoic acids, 5 flavanols, and 1 coumarin. The identified phenolic compounds, their fragmentation, and the relative standards to which they relate are listed in Table 1.

Of the 10 identified hydroxycinnamic acids, chlorogenic acid, sinapoyl hexoside derivative, cryptochlorogenic acid, *cis*-5-*O*-*p*-coumaroylquinic acid, *trans*-5-*O*-*p*-coumaroylquinic acid, caffeoyl malate, and caffeic acid derivative were already identified/determined in our previous study on *L. sativa* [5], based on their specific fragmentation patterns and the use of standards. 3,5-Diccaffeoylquinic acid was identified based on the specific fragmentation pattern of MS^2^ *m*/*z* 353, 191, and 179, and caffeoyltartaric acid was identified based on the specific fragmentation pattern of MS^2^ *m*/*z* 149, 179, and 135, as previously reported in *L. sativa* [14]. The caffeic acid derivative was identified based on the fragmentation pattern of MS^n^ ions *m*/*z* 179 and 135, as previously reported by Liu et al. [15], but was also detected for the first time in *L. sativa*. The caffeoylquinic acid dehydrodimer previously identified in *Berberis thunbergii* leaves followed its specific fragmentation pattern of MS^n^ *m*/*z* 513, 339, and 295, as previously reported by Fernández-Poyatos at al. [16].

Both hydroxybenzoic acids and one coumarin were identified in *L. sativa* in our previous study [5]. Of the five flavanols identified, kaempferol-3-*O*-glucuronoside, quercetin-3-*O*-glucoside, and quercetin-3-*O*-(6″-malonyl)-glucoside were identified by their specific fragmentation patterns and the use of standards, as previously reported by Medic et al. [5]. Quercetin-3-*O*-(6″-malonyl)-glucoside-7-*O*-glucoside and quercetin-3-*O*-(6″-malonyl)-glucoside-7-*O*-glucuronide were identified by their fragmentation pattern as reported by Abu-Reidah et al. [17] for *L. sativa*.

### 3.2. Effects of Different Composting Treatments on L. sativa Yield and Phenolic Composition

Looking at Table 2, we can see that the lowest yield for lettuce was for O + L, followed by K1 and O + L + D. The highest yield was reported for K2, O + T + D, and O + D. Interestingly, lettuce yield was higher when composting agent was added, regardless of compost type. In addition, the composting agent used also contained minerals (NPK(MgO): 4-3-1 (8)), which most likely contribute to growth and yield of treated plants. Therefore, in terms of yield, the composting agent was found to be effective in increasing the overall yield. In addition, it has been described that fresh walnut leaves still contain some allelochemicals that inhibit plant growth [13]. This is not entirely the case with the dry walnut leaves composted for 2 years, as the yield of the plants is indeed lower than with K2 (positive control) but higher than K1 (negative control), while the dry walnut leaves with the addition of composting agent have a comparable yield to K2 and higher than K1. Therefore, we suggest that dry walnut leaves and cut grass can be used for composting. Dry hazelnut leaves, on the other hand, still contain some unknown allelochemicals after 2 years that significantly and strongly inhibit plant growth and thus yield, so we would not recommend their use for composting. However, future research is needed to explain and address this problem. The higher yield of the plants with compost addition compared to K1 is related to the process of composting, as it reduces the amount of organic compounds considered harmful to the environment and reduces the carbon concentration and increases the concentration of plant nutrients, thus promoting their growth [1].

**Figure 3:**
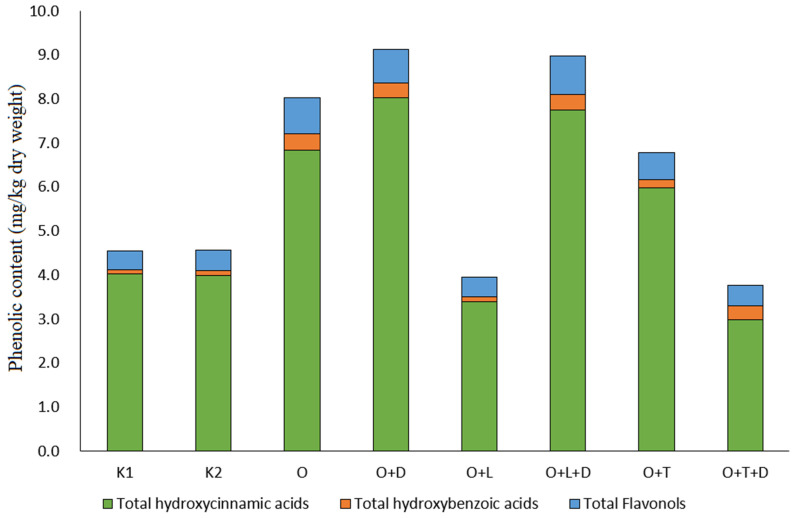
Contents of the total phenolic groups identified in *L. sativa* relative to dry weight. K1; soil without compost control, K2; soil with compost control, O; soil with composted walnut leaves, O + D; soil with composted walnut leaves with addition of composting agent, O + L; soil with composted walnut and hazelnut leaves, O + L + D; soil with composted walnut and hazelnut leaves with the addition of composting agent, O + T; soil with composted walnut leaves and grass, O + T + D; soil with composted walnut leaves and grass with the addition of composting agent.

In Table 3, we can see the individual phenolic compounds found in *L. sativa*. It can be seen that the most important phenolic compound, regardless of treatment, is chlorogenic acid, which accounts for up to 80% of the total hydroxycinnamic acids in *L. sativa*. Chlorogenic acid was followed by two flavanols (quercetin-3-*O*-(6″-malonyl)-glucoside-7-*O*-glucoside and quercetin-3-*O*-(6″-malonyl)-glucoside-7-*O*-glucuronide) and caffeoyl malate. The content of the different phenolic groups is shown in Figure 3.

Hydroxycinnamic acids were the major phenolic group in lettuce, accounting for up to 85% of TAPC (total analyzed phenolic content) (Figure 3). They were followed by flavanols and hydroxybenzoic acids, which is consistent with previous studies on phenolic compounds in lettuce [5,14,17]. Interestingly, the highest TAPC was reported for plants grown in O, O + D, and O + L compost, suggesting that some allelochemicals were still present in the compost of these treatments after two years, but in such a small amount that they did not affect yield, as the highest yield was reported for O + D, O + T + D, and K2. However, it did affect plant metabolism by stimulating the synthesis of phenolic compounds. The synthesis of phenolic compounds is related to the reaction of plants to stress conditions and is a defense mechanism of plants [18,19]. Phenolic content of plants grown in O + T + D did not differ from that of controls, indicating that the compost mixture was sufficiently composted not to interfere with plant metabolism or induce stress. Interestingly, the phenolic content of plants grown in O + L compost also did not differ from that of the controls, but the yield of these plants was the lowest, suggesting that O + L could either inhibited the growth so much that the plants metabolic synthesis was obscured as well and that higher levels of compost in the soil might even cause plant collapse. There were no differences in plant metabolism in the control treatments, suggesting that the dry walnut leaves, while affecting plant yield, also have an effect on plant metabolism by stimulating the synthesis of phenolic compounds. As previously reported, allelochemicals alter the phenolic profile and stimulate secondary metabolism in plants [5]. Since some allelochemicals can be taken up by plants and are thus toxic to consumers [20] plants were also screened for known toxic allelochemicals in walnut, i.e., juglone. However, juglone was not found in the treated plants, nor was it found in our previous study of juglone toxicity, in which we concluded that this was likely due to the fact that juglone and other naphthoquinones do not dissolve well in polar solvents and are thus also difficult to transport through the plant and might only prove problematic if the plants grown were tubers or root crops that come into direct contact with juglone [4].

Overall, walnut leaf compost does not negatively affect plant yield, but rather stimulates their growth; however, it does affect plant metabolism by stimulating the synthesis of phenolic compounds. Since phenolic compounds are considered beneficial to human health [21,22], adding walnut leaf compost to cultivated plants soil is recommended to some extent. However, as little is known about how easily these compounds leach from the soil with rainfall events, the application of walnut leaf compost should be further studied, since allelochemicals could bond with the soil and continued application could exceed the toxicity limit of the treated plant, as was the case with O + L compost, affecting not only plant metabolism but also crop yield, especially since it is known that plant phenols play an important role in plant defense against abiotic and biotic stresses [19,23]. However, to produce these valuable compounds, the plant must use nutrients and energy intended for growth and other primary functions needed to produce these defensive compounds [24,25]. The higher the content of phenolic compounds, the lower the growth of the plants, and consequently the lower the yield [5]. However, the use of hazelnut leaves for compost should be avoided until further studies are conducted to explain this phenomenon.

## 4. Conclusions

A total of 18 phenolic compounds were identified in *L. sativa*, including 10 hydroxycinnamic acids, 2 hydroxybenzoic acids, 5 flavanols, and 1 coumarin. Lettuce yield was higher when composting agent was added, regardless of compost type, indicating that composting agents are also effective. The major phenolic compound found in lettuce was chlorogenic acid, which accounted for up to 80% of the total hydroxycinnamic acids. Chlorogenic acid was followed by two flavanols (quercetin-3-*O*-(6″-malonyl)-glucoside-7-*O*-glucoside and quercetin-3-*O*-(6″-malonyl)-glucoside-7-*O*-glucuronide) and caffeoyl malate. Hydroxycinnamic acids were the major phenolic group in lettuce, accounting for up to 85% of the total analyzed phenolic content. The highest total analyzed phenolic content was found for plants grown in compost containing walnut leaves, indicating that some allelochemicals were still present in the compost of these treatments after two years, but at such a low level that they did not affect yield. Phenolic content of plants grown in compost from walnut and hazelnut leaves was also no different from controls, but yield of these plants was the lowest, suggesting that compost from walnut and hazelnut leaves could inhibit growth enough to affect plant metabolic synthesis.

We suggest that dry walnut leaves and cut grass can be used for composting, while dry hazelnut leaves, on the other hand, still contain some unknown allelochemicals after two years, which significantly and strongly inhibit plant growth and thus yield, so we would not recommend their use for composting. However, future research is needed to explain and solve this problem.

## Figures and Tables

**Table 1:** Tentative identification of the 18 phenolic compounds in *L. sativa* and the standards they are expressed to.

Compound	Rt (min)	[M−H]^−^ (*m*/*z*)	MS^2^ (*m*/*z*)	MS^3^ (*m*/*z*)	MS^4^ (*m*/*z*)	Expressed As
3,5-Dicaffeoylquinic acid	8.99	515	353 (100), 191 (74), 179 (2)			Chlorogenic acid
Dihydroxybenzoic acid hexoside 1	9.67	315	153 (100), 152 (30), 165 (14)	109 (100)		Galic acid
Dihydroxybenzoic acid hexoside 2	10.53	315	153 (100), 109 (6)	109 (100)		Galic acid
Esculetin glucoside	10.74	339	177 (100), 241 (14)	133 (100), 177 (12)		Galic acid
Caffeoylquinic acid dehydrodimer	11.71	705	513 (100), 339 (10)	339 (100), 321 (59), 469 (28), 229 (26)	229 (100), 295 (76), 293 (12)	Chlorogenic acid
Chlorogenic acid (3-caffeoylquinic acid)	13.64	353	191 (100), 179 (5)			Chlorogenic acid
Sinapoyl hexoside derivative	14.01	431	385 (100), 299 (44)			Sinapic acid
Caffeoyltartaric acid	14.30	311	149 (100), 179 (51), 135 (3)			Chlorogenic acid
Quercetin-3-*O*-(6″-malonyl)-glucoside-7-*O*-glucoside	15.24	711	667 (100)	505 (100), 463 (35), 301 (29), 462 (20)	301 (100), 300 (69)	Quercetin-3-*O*-glucoside
Cryptochlorogenic acid (4-caffeoylquinic acid)	15.49	353	191 (100), 179 (5)			Cryptochlorogenic acid
Quercetin-3-*O*-(6″-malonyl)-glucoside-7-*O*-glucuronide	16.36	725	681 (100), 505 (17)	505 (100)	301 (100), 300 (69)	Quercetin-3-*O*-glucoside
*cis*-5-*O*-*p*-coumaroylquinic acid	17.24	337	191 (100), 163 (7), 173 (1)			*p*-Coumaric acid
*trans*-5-*O*-*p*-coumaroylquinic acid	18.68	337	191 (100), 163 (7), 173 (1)			*p*-Coumaric acid
Caffeoyl malate	19.28	295	179 (100), 133 (58), 135 (5)	135 (100)		Caffeic acid
Caffeic acid derivative	21.11	415	179 (100), 225 (20), 161 (11), 143 (11)	135 (100)		Caffeic acid
Quercetin-3-*O*-glucoside	21.66	463	301 (100), 300 (17), 179 (1)			Quercetin-3-*O*-glucoside
Kaempferol-3-*O*-glucuronoside	22.47	463	287 (100), 288 (6), 151 (5)			Kaempferol-3-*O*-glucoside
Quercetin-3-*O*-(6″-malonyl)-glucoside	23.52	549	505 (100)	301 (100), 300 (69)		Quercetin-3-*O*-glucoside

Rt, retention time; [M−H]^−^, pseudo-molecular ion identified in negative ion mode; (), relative abundance of fragment ions.

**Table 2:** *L. sativa* yield measurements.

	K1	K2	O	O + D	O + L	O + L + D	O + T	O + T + D
Whole plant (g)	29.25 ± 1.18 b	81.50 ± 5.91 e	57.00 ± 6.36 d	76.00 ± 6.26 de	18.00 ± 1.58 a	37.25 ± 2.32 c	65.00 ± 4.51 d	89.75 ± 6.09 e

Data are means ± standard error. Means followed by different letters across the treatments (within rows) are significantly different (*p* < 0.05). (g), grams; K1: soil without compost control, K2: soil with compost control, O: soil with composted walnut leaves, O + D: soil with composted walnut leaves with addition of composting agent, O + L: soil with composted walnut and hazelnut leaves, O + L + D: soil with composted walnut and hazelnut leaves with the addition of composting agent, O + T: soil with composted walnut leaves and grass, O + T + D: soil with composted walnut leaves and grass with the addition of composting agent.

**Table 3:** Comparison of the content of individual phenolic compounds and phenolic groups in *L. sativa*, expressed in mg/kg dry weight.

Phenolic Compound	Quantification According to Cultivar (mg/kg Dry Weight)							
	K1	K2	O	O + D	O + L	O + L + D	O + T	O + T + D
3,5-Dicaffeoylquinic acid	0.11 ± 0.02 b	0.12 ± 0.03 b	0.18 ± 0.02 b	0.20 ± 0.00 b	0.01 ± 0.01 a	0.14 ± 0.01 b	0.11 ± 0.01 b	0.12 ± 0.03 b
Dihydroxybenzoic acid hexoside 1	0.04 ± 0.01 a	0.03 ± 0.01 a	0.03 ± 0.01 a	0.03 ± 0.02 a	0.02 ± 0.01 a	0.03 ± 0.02 a	0.03 ± 0.01 a	0.02 ± 0.00 a
Dihydroxybenzoic acid hexoside 2	0.05 ± 0.01 a	0.06 ± 0.01 a	0.30 ± 0.07 c	0.27 ± 0.04 c	0.05 ± 0.00 a	0.25 ± 0.03 bc	0.10 ± 0.03 ab	0.23 ± 0.03 bc
Esculetin glucoside	0.02 ± 0.01 a	0.02 ± 0.00 a	0.04 ± 0.01 a	0.04 ± 0.01 a	0.04 ± 0.00 a	0.08 ± 0.02 a	0.05 ± 0.02 a	0.08 ± 0.04 a
Caffeoylquinic acid dehydrodimer	0.09 ± 0.01 a	0.10 ± 0.02 a	0.11 ± 0.01 ab	0.09 ± 0.01 a	0.07 ± 0.00 a	0.10 ± 0.02 ab	0.16 ± 0.03 ab	0.19 ± 0.03 b
Chlorogenic acid (3-caffeoylquinic acid)	3.07 ± 0.11 ab	2.95 ± 0.37 a	5.40 ± 0.48 c	6.54 ± 0.62 c	2.64 ± 0.15 a	6.23 ± 0.06 c	4.87 ± 0.57 bc	2.02 ± 0.41 a
Sinapoyl hexoside derivative	0.04 ± 0.01 a	0.05 ± 0.02 a	0.02 ± 0.00 a	0.05 ± 0.01 a	0.04 ± 0.00 a	0.05 ± 0.01 a	0.03 ± 0.00 a	0.01 ± 0.00 a
Caffeoyltartaric acid	0.07 ± 0.03 a	0.07 ± 0.01 a	0.09 ± 0.01 a	0.09 ± 0.02 a	0.08 ± 0.01 a	0.11 ± 0.02 a	0.07 ± 0.01 a	0.12 ± 0.06 a
Quercetin-3-*O*-(6″-malonyl)-glucoside-7-*O*-glucoside	0.12 ± 0.05 a	0.16 ± 0.07 a	0.26 ± 0.05 a	0.34 ± 0.05 a	0.15 ± 0.02 a	0.25 ± 0.04 a	0.27 ± 0.07 a	0.15 ± 0.02 a
Cryptochlorogenic acid (4-caffeoylquinic acid)	0.13 ± 0.06 ab	0.18 ± 0.03 ac	0.25 ± 0.03 bc	0.25 ± 0.03 bc	0.09 ± 0.01 ab	0.31 ± 0.05 c	0.13 ± 0.03 ab	0.07 ± 0.04 a
Quercetin-3-*O*-(6″-malonyl)-glucoside-7-*O*-glucuronide	0.22 ± 0.02 ab	0.20 ± 0.01 a	0.35 ± 0.02 bc	0.30 ± 0.05 ac	0.22 ± 0.01 ab	0.36 ± 0.02 c	0.24 ± 0.04 ac	0.21 ± 0.02 a
*cis*-5-*O*-*p*-coumaroylquinic acid	0.17 ± 0.03 ab	0.16 ± 0.02 ab	0.26 ± 0.01 b	0.23 ± 0.04 ab	0.16 ± 0.01 ab	0.26 ± 0.02 b	0.18 ± 0.03 ab	0.13 ± 0.01 a
*trans*-5-*O*-*p*-coumaroylquinic acid	0.08 ± 0.01 a	0.09 ± 0.01 a	0.11 ± 0.01 a	0.11 ± 0.03 a	0.09 ± 0.01 a	0.12 ± 0.02 a	0.10 ± 0.02 a	0.08 ± 0.01 a
Caffeoyl malate	0.23 ± 0.04 ab	0.26 ± 0.04 ac	0.38 ± 0.02 bcd	0.44 ± 0.02 d	0.18 ± 0.03 a	0.40 ± 0.04 cd	0.31 ± 0.01 ad	0.22 ± 0.04 a
Caffeic acid derivative	0.02 ± 0.01 a	0.02 ± 0.01 a	0.02 ± 0.01 a	0.02 ± 0.01 a	0.01 ± 0.00 a	0.03 ± 0.01 a	0.02 ± 0.01 a	0.02 ± 0.01 a
Quercetin-3-*O*-glucoside	0.05 ± 0.01 a	0.04 ± 0.01 a	0.10 ± 0.03 a	0.07 ± 0.01 a	0.04 ± 0.00 a	0.08 ± 0.00 a	0.04 ± 0.01 a	0.06 ± 0.02 a
Kaempferol-3-*O*-glucuronoside	0.02 ± 0.01 a	0.02 ± 0.01 a	0.05 ± 0.01 ab	0.02 ± 0.01 a	0.02 ± 0.01 a	0.06 ± 0.01 b	0.03 ± 0.00 ab	0.02 ± 0.01 a
Quercetin-3-*O*-(6″-malonyl)-glucoside	0.02 ± 0.01 a	0.04 ± 0.04 a	0.07 ± 0.02 ab	0.03 ± 0.00 a	0.03 ± 0.01 a	0.14 ± 0.02 b	0.04 ± 0.02 a	0.02 ± 0.01 a
Total hydroxycinnamic acids	4.01 ± 0.18 a	3.99 ± 0.51 a	6.84 ± 0.50 c	8.02 ± 0.65 c	3.38 ± 0.17 a	7.74 ± 0.07 c	5.98 ± 0.60 bc	2.97 ± 0.44 a
Total hydroxybenzoic acids	0.11 ± 0.03 a	0.11 ± 0.02 a	0.37 ± 0.06 b	0.34 ± 0.05 b	0.11 ± 0.01 a	0.35 ± 0.06 b	0.18 ± 0.06 ab	0.33 ± 0.04 b
Total Flavanols	0.42 ± 0.08 a	0.46 ± 0.13 a	0.82 ± 0.08 bc	0.77 ± 0.09 b	0.46 ± 0.02 a	0.89 ± 0.04 c	0.62 ± 0.11 ab	0.46 ± 0.02 a
TAPC	4.54 ± 0.28 a	4.56 ± 0.63 a	8.02 ± 0.52 cd	9.13 ± 0.68 d	3.95 ± 0.15 a	8.99 ± 0.15 cd	6.78 ± 0.70 bc	3.76 ± 0.44 a

Data are means ± standard error. Means followed by different letters across the treatments (within rows) are significantly different (*p* < 0.05); K1: soil without compost control, K2: soil with compost control, O: soil with composted walnut leaves, O + D: soil with composted walnut leaves with addition of composting agent, O + L: soil with composted walnut and hazelnut leaves, O + L + D: soil with composted walnut and hazelnut leaves with the addition of composting agent, O + T: soil with composted walnut leaves and grass, O + T + D: soil with composted walnut leaves and grass with the addition of composting agent.

## Data Availability

The remaining data presented in this study are available on request from the corresponding author. The remaining data are not publicly available due to privacy.
